# *Notes from the Field:* Deployment of an Electronic Self-Administered Survey to Assess Human Health Effects of an Industrial Chemical Facility Fire — Winnebago County, Illinois, June–July 2021

**DOI:** 10.15585/mmwr.mm7049a4

**Published:** 2021-12-10

**Authors:** Krishna Surasi, Jasmine Y. Nakayama, Mark Johnson, Sandra Martell, Sarah Patrick, Lance R. Owen, D. Kevin Horton, Maureen Orr

**Affiliations:** ^1^Epidemic Intelligence Service, CDC; ^2^Office of Community Health and Hazard Assessment, Agency for Toxic Substances and Disease Registry, Atlanta, Georgia; ^3^Winnebago County Health Department, Rockford, Illinois; ^4^Illinois Department of Public Health; ^5^Geospatial Research, Analysis, and Services Program, Agency for Toxic Substances and Disease Registry, Atlanta, Georgia; ^6^Office of Innovation and Analytics, Agency for Toxic Substance and Disease Registry, Atlanta, Georgia.

On June 14, 2021, an industrial fluid and grease manufacturing facility in Winnebago County, Illinois, (population = 285,350) (*1*) caught fire, releasing smoke, dust, and debris for 4 days and prompting local authorities to issue a precautionary 1-mile (1.5-km) evacuation order and 3-mile (5-km) masking advisory around the location of the facility during this time. Review of Electronic Surveillance System for the Early Notification of Community-based Epidemics (ESSENCE) data during this time demonstrated increased emergency department visits in five zip codes downwind of the fire. In response, the Winnebago County Health Department (WCHD), Illinois Department of Public Health, and Agency for Toxic Substances and Disease Registry (ATSDR) collaborated to investigate the fire’s effect on human health.

ATSDR offers epidemiologic assistance to state and local public health authorities after chemical incidents through Assessment of Chemical Exposure (ACE) investigations. These investigations might use ACE and Epidemiologic Contact Assessment Symptom Exposures toolkits, which include interviewer-administered health surveys that can be quickly modified to collect relevant information (e.g., exposure and symptom data) to guide response and recovery efforts (*2*,*3*). For this investigation, these surveys were combined and adapted into a single, electronic, self-administered survey to facilitate rapid and wide distribution.

As a public health authority responsible for assessing public health events, WCHD used an existing electronic system that had previously been used for COVID-19 vaccination registration to distribute the survey by email. Survey links were emailed to all persons registered in this electronic system who had a valid email address and who resided in 11 selected zip codes (the five identified by ESSENCE data plus six additional zip codes nearby [total population = 247,059]) (*4*). This electronic system allowed only one survey to be submitted per emailed link during July 5–15, 2021. WCHD also promoted survey completion through door-to-door flyer distribution, news outlets, social media, and their own website that included a different link which could be used to submit multiple surveys during July 1–15, 2021. Geospatial analyses were performed at the U.S. Census tract level with ArcGIS Pro (version 2.8.2; Esri) to assess geographic distribution of survey respondents’ reported home addresses and symptoms. Home addresses from the survey were geocoded and then joined to demographic data from the 2019 American Community Survey to calculate response rates (*5*).

Among 40,217 survey links emailed through the electronic system, 1,807 (4.5%) were accessed to submit a survey. An additional 223 surveys were received from links accessed on WCHD’s website or social media, for a total of 2,030 unique survey respondents. Most respondents were White persons (1,754; 86.4%), not Hispanic or Latino persons (1,928; 95.0%), and female (1,277; 62.9%). Mean age was 50 years (range = 11–94 years). Among respondents, 916 (45.1%) reported one or more new or worsened symptom since the fire, typically related to the ears, nose, and throat (638; 69.7%); nervous system (478; 52.2%); and eyes (383; 41.8%). Four respondents reported having been hospitalized. The highest survey response rate (37.9 surveys per 1,000 residents) was from the U.S. Census tract where the facility was located (Figure); that tract also included the highest percentage of survey respondents reporting any symptom (154 of 241; 63.9%).

**FIGURE:**
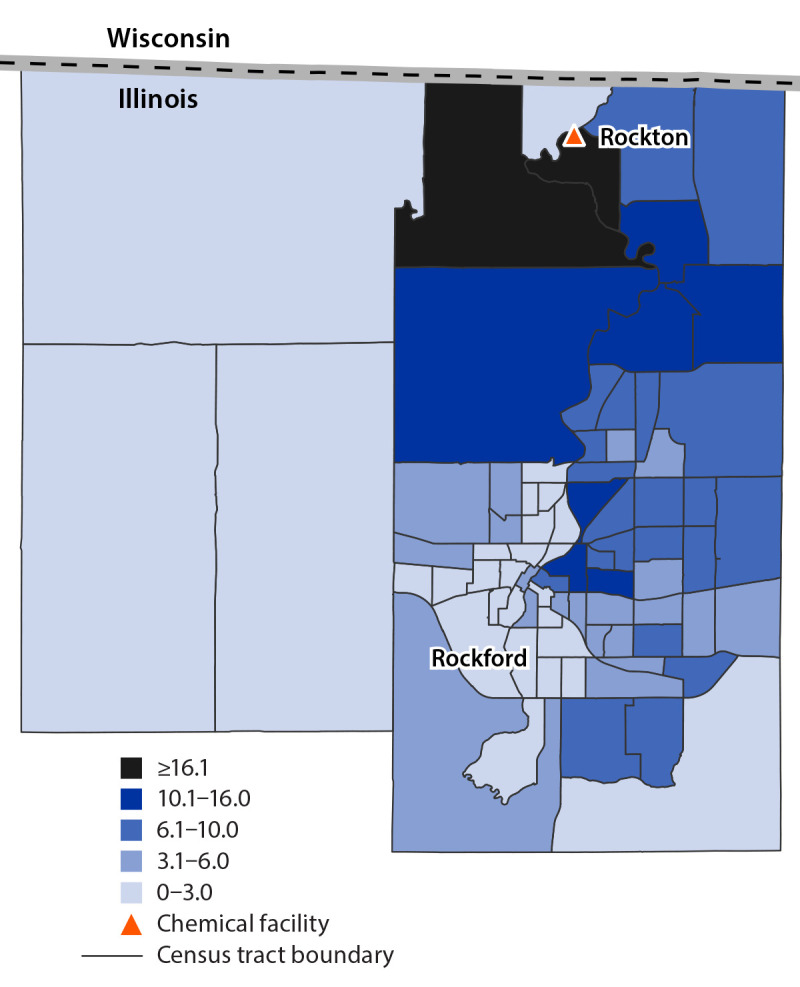
Human health survey completion rate per 1,000 residents after a chemical manufacturing facility fire, by U.S. Census tract — Winnebago County, Illinois, July 1–15, 2021* * Data from Winnebago County Health Department (health survey data responses and locations), U.S. Census Bureau American Community Survey 2019 5-year estimate (population of U.S. Census tracts), Esri (geometry of U.S. Census tracts), and Agency for Toxic Substances and Disease Registry (location of chemical facility).

Survey distribution through the electronic system enabled enrollment of approximately twice as many survey respondents than that in previously reported ACE investigations (*2*). The electronic system also facilitated sending targeted follow-up questions to only those respondents whose initial survey answers indicated that they could provide additional relevant information. Geospatial analyses allowed assessment of reported home addresses and symptoms among respondents, thereby enabling rapid and focused adjustments during the survey period, including promoting the survey with informational flyers in an area close to the facility with a low response rate that was identified by geospatial mapping.

This was the first documented use of an electronic, self-administered survey in an ACE investigation. One limitation was the use of a convenience sample, mostly consisting of persons registered for the electronic COVID-19 vaccination registration system. Respondents using this system might be more comfortable with electronic communications and interested in public health activities than is the overall affected population. Also, a low response rate to the emailed survey link was reported. However, future ACE investigations might benefit from this approach, which permits efficient surveying in a wide geographic distribution after a chemical incident. In addition, this response highlights how data modernization–driven public health resources developed during the COVID-19 pandemic can be adapted to serve other public health needs.
